# Therapeutic Potential of Peptides in Cancer Treatment: Focus on Peptide and Aptamer-Decorated Exosomes

**DOI:** 10.3390/cancers18081214

**Published:** 2026-04-10

**Authors:** Prakash Gangadaran, Aswini Suresh Kumar, Kasinathan Kumaran, Kruthika Prakash, Sanjana Dhayalan, Ramya Lakshmi Rajendran, Vasanth Kanth Thasma Loganathbabu, Janani Balaji, Radhika Baskaran, Raksa Arun, Vanshikaa Karthikeyan, Sreyee Biswas, Chae Moon Hong, Kandasamy Nagarajan ArulJothi, Byeong-Cheol Ahn

**Affiliations:** 1 Department of Nuclear Medicine, School of Medicine, Kyungpook National University, Daegu 41944, Republic of Korea; prakashg@knu.ac.kr (P.G.); ramyag@knu.ac.kr (R.L.R.); cmhong@knu.ac.kr (C.M.H.); 2 Cardiovascular Research Institute, Kyungpook National University, Daegu 41944, Republic of Korea; 3 Department of Genetic Engineering, Faculty of Engineering and Technology, SRM Institute of Science and Technology, Kattankulathur, Chengalpattu 603203, India; as0736@srmist.edu.in (A.S.K.); kk3009@srmist.edu.in (K.K.); kp8418@srmist.edu.in (K.P.); sd2312@srmist.edu.in (S.D.); vt7848@srmist.edu.in (V.K.T.L.); jb9929@srmist.edu.in (J.B.); rb7340@srmist.edu.in (R.B.); ra3908@srmist.edu.in (R.A.); vk6193@srmist.edu.in (V.K.); 4 BK21 FOUR KNU Convergence Educational Program of Biomedical Sciences for Creative Future Talents, School of Medicine, Kyungpook National University, Daegu 41944, Republic of Korea; 5 Department of Biotechnology and Medical Engineering, National Institute of Technology Rourkela, Rourkela 769008, India; 225bm2006@nitrkl.ac.in; 6 Department of Nuclear Medicine, Kyungpook National University Hospital, Daegu 41944, Republic of Korea

**Keywords:** peptides, aptamers, exosomes, precision oncology, ligand-targeted therapy

## Abstract

Traditional cancer therapies such as surgery, chemotherapy, and antibody-based treatments often face significant barriers, including systemic toxicity, a lack of selectivity, and the emergence of drug resistance. These issues demand innovative and targeted solutions. Peptide-based therapeutics have gained prominence for their ability to disrupt cancer pathways and facilitate targeted drug delivery, offering structural flexibility, precise targeting, and low immunogenicity with minimal effects on healthy tissues. Concurrently, aptamers, which are structured nucleic acid molecules capable of high-affinity molecular recognition, are being developed as both direct therapeutic agents and as targeting ligands for the improved delivery of anticancer drugs. Combining peptide and aptamer technologies with engineered exosomes provides a modular drug delivery system that enhances targeting specificity, stability, and the ability to cross complex biological barriers such as the blood–brain barrier. The emergence of peptide-decorated, aptamer-decorated exosomes represents a new frontier in precision oncology, promising highly selective, biocompatible, and tunable cancer therapies. Further advances are required to overcome challenges in pharmacokinetics, scalable production, and regulatory compliance, but ongoing bioengineering and nanotechnology research continues to accelerate the translation of these innovative strategies toward improved cancer diagnostics and treatment outcomes. This review discusses the synergistic integration of peptides and aptamers with exosome-based delivery systems, highlighting their current applications and future possibilities.

## Simple Summary

Cancer treatment is often constrained by side effects, limited selectivity, and drug resistance. Peptides and aptamers are small molecules capable of recognizing cancer-related targets with high specificity, while exosomes are natural carriers that can transport therapeutic agents throughout the body with excellent biocompatibility. This review highlights how exosomes decorated with peptides and aptamers may enhance the selective delivery of anticancer drugs, improve tumor targeting, and minimize damage to healthy tissues. We summarize recent advances in peptide therapeutics, aptamer-based targeting, and exosome engineering, and discuss their combined potential as a next-generation platform for safer, more effective, and more personalized cancer treatment.

## 1. Background

Over the last decade, conventional anticancer treatments, such as surgery, ionizing radiation, cytotoxic chemotherapy, and various immunotherapeutic and antibody-based approaches, have remained core pillars of cancer therapy. More recently, Chimeric Antigen Receptor T-cell (CAR-T) therapy has emerged as a state-of-the-art treatment strategy. CAR-T therapy involves genetically engineered patient-derived T cells to recognize tumor-specific antigens, enabling precise targeting and producing remarkable clinical responses in certain hematological malignancies. Despite these advances, the therapeutic potential of current approaches is frequently constrained by practical limitations including systemic toxicity, poor selectivity, treatment resistance, and limited efficacy in solid tumors [1,2]. These limitations emphasize the urgent need to develop innovative therapeutic strategies that can address the shortcomings of current treatments, including their lack of selectivity. This necessitates exploring peptide-based therapeutics as a promising next-generation approach for safer and more targeted cancer therapy.

Peptide-based therapies have rapidly gained attention in cancer research as a new class of drugs that bridge the gap between small chemical drugs and large protein biologics. Their high target specificity, low immunogenicity, and varied design potential make them excellent candidates for precision oncology [3]. In addition, the expanding global peptide therapeutics market reflects growing confidence in peptide-based medicines, with an increasing number of peptide drugs entering clinical trials and receiving regulatory approval. Peptides can be designed to recognize tumor-specific receptors, disrupt essential signaling pathways, or serve as drug and nucleic acid transporters, providing targeted delivery with minimal damage to healthy tissues.

Their ease of synthesis and chemical tunability enable rapid optimization through the incorporation of non-natural amino acids and cyclization to enhance stability and binding affinity. Despite these advantages, the therapeutic application of peptides faces significant challenges. Proteolytic degradation, short plasma half-life, and rapid renal clearance reduce their bioavailability [4].

In parallel with advances in peptide therapies, another promising approach involves the use of aptamers, which are short, single-stranded DNA or RNA oligonucleotides that fold into unique three-dimensional structures capable of binding specific targets with antibody-like accuracy [5]. Aptamers, like peptides, exhibit excellent selectivity, minimal immunogenicity, and tunable chemistry, making them practical tools for targeted cancer treatment. Their synthetic adaptability allows for simple modifications to increase stability, associate with medicines, or attach to nanocarriers for improved delivery [6]. Importantly, aptamers can function as both therapeutic agents (blocking disease-related targets) and ligands [5]. As molecular breakthroughs advance, the decorated roles of peptides and aptamers are converging, ushering in a new era of precision-engineered cancer therapies. 

Although peptides and aptamers offer effective and precise targeting capabilities, their short circulation half-life, quick enzymatic breakdown, and low stability in biological settings frequently restrict their direct therapeutic application. Exosome-based delivery methods have drawn more interest as a physiologically suitable platform for targeted therapy in order to overcome these difficulties. Exosomes are naturally occurring extracellular vesicles (EVs) with intrinsic membrane characteristics that can prolong systemic circulation, improve cellular absorption, and shield encapsulated cargo from destruction. Crucially, peptides or aptamers can be added to the surface of exosomes to combine effective transport with accurate molecular targeting. By increasing specificity, stability, and bioavailability, this integrated method improves the overall therapeutic potential, demonstrating that exosomes serve as enabling systems that boost the effectiveness of peptide- and aptamer-based treatments rather than as redundant components [7,8].

However, their manufacture is still complicated and expensive. Peptides and aptamers have become viable complementary targeting ligands in this setting. Better tissue penetration and quick binding kinetics are made possible by their reduced size, while easy chemical modification and economical manufacture are made possible by their synthetic accessibility. Aptamers also have a high binding affinity that is similar to that of antibodies, however they are frequently less immunogenic. However, it is crucial to remember that these compounds are positioned as supplementary or alternative tools that may be useful in particular therapeutic and diagnostic contexts rather than as antibody substitutes [9,10].

The combination of their distinct strengths, peptides with their receptor-targeting efficiency and aptamers with their structural adaptability, creates new opportunities for developing innovative, biocompatible systems capable of entering the complicated TME. Another emerging approach in this field involves utilizing exosome-based delivery systems [11,12]. Exosomes are endogenous nanovesicles secreted by cells that serve as natural carriers of proteins, lipids, and nucleic acids, thus facilitating intercellular communication and targeted molecular transfer [13]. Exploring these interrelated techniques offers a glimpse into the future of oncology, where treatments are not only more effective but also intelligently designed to work in harmony with biological processes rather than disrupt them [14]. 

## 2. Peptides in Cancer Therapy

Therapeutic peptides consist of well-ordered amino acids, typically with molecular weights ranging from 500 to 5000 Da. Since the synthesis of the first therapeutic peptide, insulin, the approval of additional peptide drugs has been initiated [15,16].

### 2.1. Classification 

**Figure 1:**
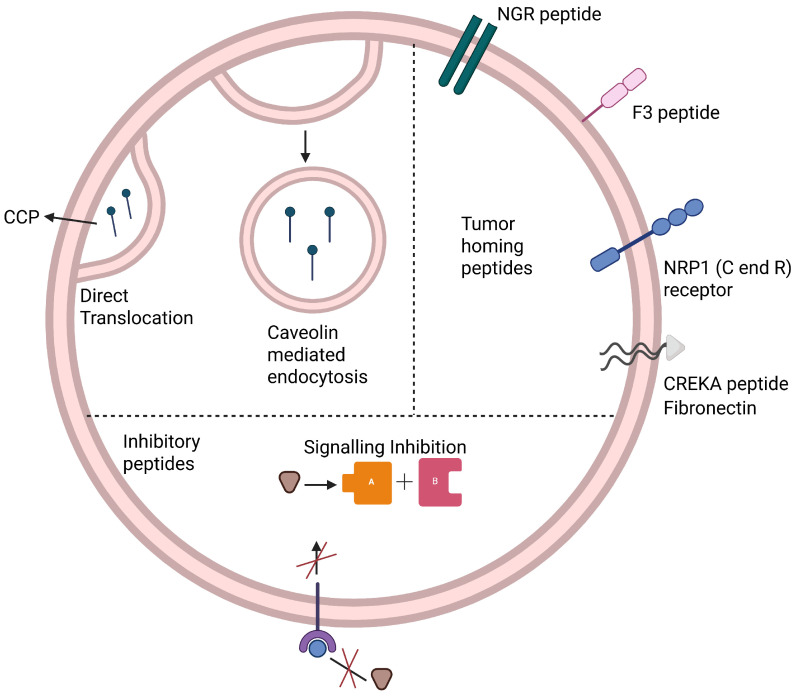
Peptide Classification. Tumor-homing peptides (NGR, F3, CREKA) target receptors or extracellular matrix components, while cell-penetrating peptides enter cells via direct translocation or endocytosis. Inhibitory peptides block oncogenic signaling pathways (A, B represent the downstream signaling). CCP: Clathrin-coated pit; NGR: Asn-Gly-Arg; F3: Tumor-homing peptide; NRP1: Neuropilin-1; CREKA: Cys-Arg-Glu-Lys-Ala. Created in BioRender. Gangadaran, P. (2026) https://BioRender.com/a2sv97k, accessed on 6 April 2026.

Based on their mode of action, peptides can be classified as cell-penetrating, tumor-homing, and inhibitory peptides. As illustrated in Figure 1, these peptide classes differ not only in function but also in their mechanisms of cellular interaction, with cell-penetrating peptides facilitating intracellular delivery, tumor-homing peptides enabling receptor-specific targeting, and inhibitory peptides directly modulating oncogenic pathways.

#### 2.1.1. Cell-Penetrating Peptides (CPPs)

CPPs are short (<30 amino acids) and can cross the cell membrane, delivering cargo efficiently to cancer cells even at low concentrations [17,18].

They can be conjugated to macromolecules such as DNA, RNA, and proteins, facilitating their transport across otherwise impermeable cell membranes [19]. Cationic CPPs, particularly those with arginine-rich sequences, possess a net positive charge at physiological pH, allowing for direct penetration of cell membranes without receptor mediation. Uptake efficiency increases with arginine length up to 8–10 residues, after which membrane damage may occur [20]. In HeLa cells, the cationic cell-penetrating peptide R9 (nona-arginine) primarily enters through endocytosis at low concentrations. However, at higher concentrations, R9 triggers activation of sphingomyelinase, which hydrolyzes sphingomyelin into ceramide, thereby altering membrane composition and enabling direct membrane penetration [21].

Anionic CPPs are peptides with a net negative charge. p28, a 28-amino acid fragment of the azurin protein from *Pseudomonas aeruginosa*, selectively permeates cancer cells. Originally known as an electron transfer protein, azurin has gained attention for its ability to target cancer. The p28 region forms an α-helix and binds lipid rafts—membrane microdomains overexpressed in cancer cells—to mediate selective cellular entry [22]. Amphipathic CPPs, containing both polar and nonpolar residues, penetrate cell membranes by interacting with lipid components, making them the most common CPP type. pVEC, a CPP sequence (LLIILRRRIRKQAHAHSK-NH_2_), efficiently crosses cell membranes. Researchers conjugated it with a glioma-homing peptide (gHo: NHQQQNPHQPPM-NH_2_) to specifically target glioma cells. This hybrid peptide was conjugated with a fluorescent tag (FAM), enabling targeted delivery of the fluorescence marker directly into glioma cells, combining membrane penetration with precise tumor localization [23].

#### 2.1.2. Tumor-Homing Peptides

These peptides selectively enter tumor cells by binding to overexpressed surface receptors or receptor-associated proteins, followed by endocytosis. Unlike conventional CPPs, which broadly penetrate various cells, these peptides target specific tumor or TME cells, often without affecting the function of normal cells [24].

**Figure 2:**
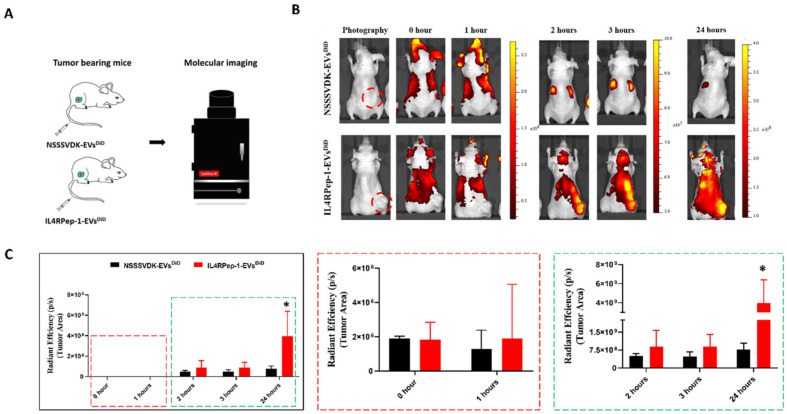
Intravenously injected IL4RPep-1–EVs selectively target Cal-62 tumors in mice. (**A**) Schematic of Cal-62 tumor-bearing mice receiving NSSSVDK-EVDiD or IL4RPep-1-EVDiD (50 μg EVs) followed by IVIS imaging. (**B**) Fluorescent images at 0, 1, 2, 3, and 24 h post-injection (n = 3). (**C**) Quantified tumor-region fluorescence (mean ± SD). * represent statistical analysis by Student’s *t*-test; *p* < 0.05. (Adapted with permission from Gangadaran et al. [18]; Copyright © 2021, The Author(s), CC-BY 4.0 International License).

A recent study introduced a novel targeted drug delivery approach using interleukin-4 receptor (IL-4R)-binding peptide (IL4RPep-1)-decorated EVs derived from mesenchymal stem cells. Through dioleylphosphatidylethanolamine-based anchoring, EVs achieved ~99.9% decoration efficiency without structural alteration. The engineered EVs showed enhanced uptake in IL-4R-expressing anaplastic thyroid cancer (Cal-62) cells and selective tumor accumulation within 2 hours post-injection in mice. This non-genetic modification strategy offers a clinically translatable platform for precise, receptor-specific EV-based therapy, minimizing off-target effects in IL-4R-positive cancers (Figure 2) [25]. As shown in Figure 2, the rapid and selective tumor accumulation highlights the efficiency of peptide-mediated targeting and supports its translational potential for receptor-specific delivery.

One well-studied example is iRGD (sequence: CRGDKGPDC), a cyclic peptide that targets tumors. It first binds to αvβ3 integrin, a receptor highly expressed on tumor cells and tumor-associated endothelial cells. Proteolytic cleavage then exposes a C-end Rule (CendR) motif, enabling binding to neuropilin-1 (NRP-1). These dual bindings trigger endocytosis, allowing iRGD to penetrate tumor tissues deeply. Beyond delivery, iRGD can inhibit cancer metastasis in vivo and enhance the uptake of co-administered drugs specifically into tumor sites, making it both a targeting and therapeutic agent [26]. The SP5-52 peptide binds selectively to tumor neovasculature through its unique proline–serine–proline (PSP) consensus motif, which is critical for molecular recognition. Binding occurs via high-affinity interactions between the PSP motif and tumor endothelium-associated markers induced by vascular endothelial growth factor (VEGF) signaling [27].

#### 2.1.3. Inhibitory Peptides

Inhibitory peptides are short amino acid sequences that block or disrupt specific biological processes, usually by interfering with protein–protein, protein–DNA, or protein–receptor interactions. They often mimic a natural binding region of one protein, allowing them to bind to a target and competitively prevent its normal function [28].

ATSP-7041, a stapled α-helical inhibitory peptide, is designed to restore the tumor suppressor p53 in cancers with wild-type p53 that MDM2 and MDMX inactivate. ATSP-7041 mimics p53’s binding domain but is chemically “stapled” to maintain its α-helical shape. By binding tightly to MDM2 and MDMX, it prevents them from degrading or inhibiting p53, thereby reactivating p53’s tumor-suppressive functions, such as cell cycle arrest and apoptosis [29]. The P17 peptide functions as a specific inhibitor of tumor growth factor (TGF)-β1, blocking its interaction with receptors and downstream signaling. By suppressing TGF-β1-induced extracellular matrix synthesis, including collagen and fibronectin production, P17 effectively reduces peritoneal fibrosis and limits the formation of a tumor-supportive stroma. In gastric cancer, this inhibition disrupts cancer–mesothelial cell crosstalk, thereby reducing tumor adhesion, invasion, and peritoneal metastasis [30].

Table 1 summarizes the types and mechanisms of action of representative therapeutic peptides in cancer, including cell-penetrating, tumor-homing, and inhibitory peptides involved in targeted drug delivery and apoptosis induction.

### 2.2. Peptide Drug Design and Modifications

Peptide therapeutics face two major hurdles: poor membrane permeability due to their size, hydrophilicity, and hydrogen-bonding profile. And also, they low in vivo stability resulting from the rapid enzymatic cleavage of their flexible amide backbones and the lack of stabilizing secondary and tertiary structures. These drawbacks lead to short half-lives and rapid elimination. Structural modifications have become key strategies to improve bioavailability, selectivity, and metabolic resilience [42].

#### 2.2.1. Backbone Modification

Backbone modification alters the peptide’s main chain (amide backbone) to enhance stability, bioavailability, and resistance to enzymatic degradation. It modifies the fundamental peptide bonds rather than side chains. Standard methods include substituting L-amino acids with D-amino acids, introducing N-alkylated or β-amino acids, and replacing amide bonds with isosteric mimics. For instance, replacing L-amino acids with their D-forms prevents protease recognition, significantly increasing peptide half-life. This principle is also used in lanthipeptides, where D-amino acids are enzymatically introduced to confer remarkable stability [43]. Another example is peptoids, which shift the side-chain position from the α-carbon to the nitrogen atom (N-substituted glycines). This alteration eliminates hydrogen-bond donors and reduces the protease susceptibility [42,44].

#### 2.2.2. Side Chain Modification

Side chain modification involves altering amino acid side groups to enhance solubility, affinity, and resistance to oxidation. A notable example is octreotide, a somatostatin analog in which unnatural amino acids (such as D-tryptophan and threoninol) stabilize the peptide, increasing receptor binding and half-life compared to the native somatostatin [45]. Buserelin, a potent synthetic gonadotropin-releasing hormone analog, enhances stability and potency through glycine-to-D-serine substitution and C-terminal butylamide modification. Prolonged use suppresses pituitary gonadotropins, effectively inducing reversible medical castration for prostate cancer [46].

#### 2.2.3. Peptide Cyclization

Peptide cyclization enhances conformational stability, proteolytic resistance, and receptor-binding affinity, making it a valuable strategy in the design of anticancer drugs. For example, cyclized p53-MDM2 inhibitory peptides structurally constrain α-helical motifs, thereby restoring p53 activity by competitively blocking MDM2 interaction and leading to apoptosis in tumors expressing wild-type p53 [47]. The cyclic somatostatin analog TT-232 exhibits high affinity for tumor-specific somatostatin receptor subtypes, inducing caspase-mediated apoptosis [48].

#### 2.2.4. Terminal Modifications

Terminal modification is a crucial structural optimization strategy where the N- or C-terminus of a peptide is chemically altered to enhance proteolytic resistance, plasma stability, and receptor affinity. For example, bortezomib, a C-terminally modified dipeptidyl boronic acid, inhibits the 26S proteasome by forming a reversible covalent bond with its catalytic threonine residue, leading to apoptosis in multiple myeloma cells [49].

## 3. Aptamers as Therapeutic Tools

With the advent of systematic evolution of ligands by exponential enrichment (SELEX), ligand discovery was revolutionized, allowing the screening of vast libraries of nucleic acids for high-affinity binders termed aptamers [50].

### 3.1. Introduction to Aptamers

Aptamers are short, single-stranded oligonucleotides (DNA or RNA) or, less commonly, peptide molecules that can fold into unique three-dimensional conformations, binding with high affinity and specificity to specific targets [51]. With the discovery of the SELEX process, molecular recognition technology was revolutionized by enabling the selection of ligands against any target, including proteins, small molecules, and even whole cells. Although aptamers are widely used as delivery systems, they structurally mimic the binding characteristics of antibodies. They are often referred to as chemical antibodies but offer several advantages, such as lower immunogenicity, smaller size (~5–15 kDa), chemical stability, and ease of synthesis and modification. Unlike antibodies, which require animal hosts for production, aptamers are generated entirely in vitro, allowing for precise control over sequence optimization [5].

Moreover, aptamers can form various secondary and tertiary structures, such as G-quadruplexes, stem-loops, hairpins, and pseudoknots, which confer high structural diversity. This enable them to interact with diverse molecular surfaces, similar to how peptides form secondary motifs such as α-helices and β-sheets for target binding. This structural versatility underlies their ability to function analogously to peptides, enabling them to replace or enhance peptide-based targeting and therapeutic systems in oncology.

### 3.2. Aptamers in Cancer Diagnosis and Therapy

Aptamers have emerged as multifunctional tools for both cancer diagnosis and therapy, acting as targeting ligands, receptor antagonists, and delivery vehicles. Their high binding affinity (typically in the low nanomolar to picomolar range) allows them to precisely recognize tumor-specific antigens, growth factor receptors, or other surface markers [52].

Mechanistically, aptamers function through several key modalities. They can specifically bind to overexpressed cell surface proteins such as VEGF, endothelial growth factor receptor (EGFR), human epidermal growth factor receptor 2 (HER2), and nucleolin, inhibiting receptor-mediated signaling pathways involved in tumor progression. For example, VEGF-targeting aptamers have shown efficacy in disrupting angiogenic signaling cascades, thereby suppressing tumor neovascularization [53]. Specific aptamers undergo receptor-mediated endocytosis upon binding to their target, facilitating the intracellular delivery of conjugated therapeutic agents, including small molecules, peptides, small interfering RNAs (siRNAs), or nanoparticles [54]. Aptamers can be coupled with imaging probes (e.g., fluorophores, magnetic resonance imaging contrast agents, radionuclides) to enable simultaneous diagnosis and therapy (theranostics), offering non-invasive monitoring of drug biodistribution and therapeutic response [55,56,57,58]. The performance of aptamers can be improved through chemical base modifications that enhance nuclease resistance, binding affinity, and pharmacokinetic stability. Modified nucleotides such as benzyl-deoxyuridine (BzdU), naphthyl-deoxyuridine(NpdU), 2’-fluro(2’-F) substitutions, and 2’-O-methyl(2’-Ome) modification have widely incorporated during aptamer selection to generate highly stable variants with improved target interaction. These modifications can mimic amino acid side chains, increase hydrophobic interaction with protein targets, and significantly enhance the resistance to enzymatic degradation to biological fluids [59,60]. Some aptamers have also been shown to modulate immune responses by targeting immune checkpoints or cytokine receptors such as PD-1 (programmed cell death protein 1), PD-L1 (programmed death-ligand 1), CTLA-4 (cytotoxic T-lymphocyte-associated protein 4), TIM-3 (T-cell immunoglobulin and mucin-domain containing-3), LAG-3 (lymphocyte-activation gene 3), and TIGIT (T-cell immunoreceptor with immunoglobulin and immunoreceptor tyrosine-based inhibition motif domains). This opens new avenues for immunotherapeutic applications in cancer treatment [61]. Collectively, these functions position aptamers as dual-function molecules, serving as both recognition elements and therapeutic agents in precision oncology.

#### 3.2.1. AS1411 Aptamer as a Model System

Among the numerous aptamers investigated for oncological applications, AS1411 stands out as a prototypical DNA aptamer that has advanced through multiple stages of preclinical and clinical evaluation. AS1411 forms a G-quadruplex structure and specifically targets nucleolin, a multifunctional protein overexpressed on the surface of various cancer cells but minimally expressed on normal cells [62]. AS1411 binds to surface-expressed nucleolin, inducing receptor-mediated internalization in cancer cells. Once internalized, it can interfere with nucleolin’s regulatory functions in mRNA stabilization, ribosome biogenesis, and cell proliferation [63]. Furthermore, AS1411 has been utilized as a targeting moiety in drug conjugates and nanocarriers, facilitating the site-specific delivery of chemotherapeutics such as doxorubicin, cisplatin, and paclitaxel [62]. AS1411-conjugated nanoparticles and liposomes have also demonstrated enhanced tumor accumulation and reduced off-target distribution. When combined with other therapeutic agents or aptamers, AS1411 improves cellular uptake and synergistically enhances anticancer efficacy. The success of AS1411 underscores the feasibility of aptamers as clinically translatable functional analogs to peptides, bridging molecular recognition and therapeutic action [64,65].

#### 3.2.2. Sgc8c Aptamer

Another well-characterized example is the Sgc8c aptamer, originally selected against CCRFCEM T-cell leukemia cells via cell-SELEX. Sgc8c recognizes protein tyrosine kinase 7 (PTK7), a membrane receptor implicated in Wnt signaling and tumor progression [66]. This aptamer exhibits high selectivity and internalization efficiency, making it an ideal candidate for targeted delivery applications in hematologic and solid malignancies. Upon binding PTK7, Sgc8c undergoes endocytosis, enabling intracellular transport of conjugated therapeutics. It has been employed in the design of aptamer–drug conjugates, aptamer-decorated nanoparticles, and aptamer–siRNA complexes, each demonstrating efficient cellular uptake and potent antitumor activity in PTK7-positive cancer models [67].

SGC8c–doxorubicin conjugates have shown significant cytotoxicity against leukemia cells while sparing normal cells [68]. SGC8c has been integrated into electrochemical and fluorescence biosensors for early detection of leukemia [68,69]. The success of Sgc8c highlights the versatility of aptamers in precision-targeted therapy and reinforces their role as functional analogs of therapeutic peptides in receptor-specific tumor targeting.

### 3.3. Limitations and Challenges of Aptamers

While aptamers have shown strong preclinical efficacy, their clinical translation is still limited by several pharmacological and logistical barriers. Aptamers are susceptible to nuclease degradation and rapid renal clearance due to their small size. Translational approaches include PEGylation, lipid conjugation, and encapsulation within exosomes or nanoparticles, which collectively enhance serum half-life and bioavailability. Tumor heterogeneity can reduce the efficacy of single-target aptamers. Dual-targeting aptamers or aptamer–peptide conjugates offer a strategy to circumvent this by simultaneously engaging multiple receptors or tumor-associated epitopes. Standardized protocols for aptamer synthesis, purification, and quality control are still under development. For translational adoption, good manufacturing practice-compliant scalable manufacturing and validated pharmacodynamic endpoints are essential. Aptamers often accumulate in endosomes post-internalization [70].

## 4. Exosomes as Natural Nanocarriers in Cancer Therapy

### 4.1. Introduction to Exosomes

Exosomes are single-membrane, lipid-bilayer EVs measuring 30–150 nm in diameter. They are produced by the inward budding of the endosomal membrane, maturing into multivesicular bodies that fuse with the plasma membrane, releasing the exosome into the extracellular space. Exosomes are secreted by all living cells and are found in various body fluids, including saliva, plasma, semen, tears, urine, and breast milk. The essential components of exosomes include nucleic acids (DNA, RNA, mRNA), lipids, and numerous proteins, such as receptors, enzymes, and transcription factors.

Exosomes carry specific molecular information from parental cells, functioning as a means of intercellular communication that facilitates the transfer of functional proteins, lipids, mRNAs, and microRNAs (miRNAs) over long distances. This information can assist in the diagnosis and treatment of a wide range of diseases, including inflammatory, cancerous, degenerative, and cardiovascular conditions.

They are highly stable and can transport drug molecules over long distances, facilitating drug delivery in various physiological and pathological conditions [71]. Exosomes can be derived from various cell types, such as stem cells, immune cells, and tumor cells, each exhibiting different compositions. These differences result in significant alterations in their immunogenicity, biological function, and therapeutic applications. Despite these variations, exosomes express surface markers such as CD63 and CD81, which are used for their characterization and isolation.

Exosomes can be isolated using various methods, including ultrafiltration, size exclusion chromatography, immunoaffinity capture, and ultracentrifugation. Each technique varies in scalability, yield, and purity. Cancer-derived exosomes contain diverse molecular cargo, including oncogenes and biomarkers. This cargo can be analyzed using chipsets and immunoaffinity techniques for early cancer diagnosis.

Exosome biogenesis plays a crucial role in therapeutics, including exosome-based nanovaccines. Scientists genetically modify exosomes to incorporate tumor-derived antigenic material, thereby creating personalized vaccines against cancer. Exosomes interact with recipient cells through multiple internalization processes, such as phagocytosis, clathrin-mediated endocytosis, and direct membrane fusion [72].

### 4.2. Exosome Engineering Strategies

Exosomes can be engineered using two primary methods: drug loading and surface modifications.

#### 4.2.1. Drug Loading

Exosomes can be loaded with artificial nanoparticles and low-molecular-weight therapeutic agents for combination therapy. This drug loading can be categorized as either endogenous or exogenous. Endogenous loading involves loading cargo into parental cells, which then incorporate the cargo into multivesicular bodies during exosome biogenesis, ultimately releasing cargo-loaded exosomes into the extracellular space. Exogenous loading is the process of loading cargo into exosomes by exploiting the dynamic nature of the lipid bilayer. This approach includes passive and active loading. Passive loading involves incubating the cargo and exosomes at suitable temperatures and pH levels, resulting in encapsulation through diffusion. For example, colorectal tumors can be effectively targeted using aptamer-decorated exosomes loaded with doxorubicin [8]. Active loading utilizes physical or chemical methods to enhance membrane permeability, resulting in encapsulation. Active methods alter membrane structure, enabling the encapsulation of hydrophilic and macromolecular cargo.

#### 4.2.2. Surface Modifications

Bioengineering methods can modify the surface of exosomes and can be classified into three categories: chemical, physical, and genetic modifications. Chemical modifications include both covalent and noncovalent modifications. Covalent modifications, such as click chemistry and PEGylation, help provide stability when attaching ligands and polymers. Through click chemistry, a neuropilin-1-targeted peptide was conjugated with exosomes using sulfonyl azide. This system crossed the BBB and effectively targeted the tumor area for glioma treatment. Noncovalent modifications utilize electrostatic interactions, receptor-ligand binding, and hydrophobic insertion to modify exosomal membranes. 

Physical modifications involve utilizing physical forces to alter the surface of exosomes. Several physical modification methods, such as natural incubation, facilitate membrane fusion through electrostatic or hydrophobic interactions. Hybridization increases exosome size, improving drug loading efficiency and allowing for the incorporation of macromolecular cargo. Gomes et al. fused pH-sensitive, long-circulating liposomes containing doxorubicin to breast cancer tumor exosomes for breast cancer treatment. Genetic modifications can promote site-specific insertion, modification, and deletion at specific genomic sites, leading to the production of higher-quality exosomes. Bai et al. transfected HEK293T cells with an engineered tLyp-1-lamp2b plasmid to obtain tumor-specific tLyp-1-expressing exosomes that encapsulate siRNA. These siRNA tLyp-1 exosomes were used to reduce the stemness of cancer stem cells by targeting stemness-associated genes [72].

## 5. Synergistic Role of Aptamers and Peptide-Decorated Exosomes

**Figure 3:**
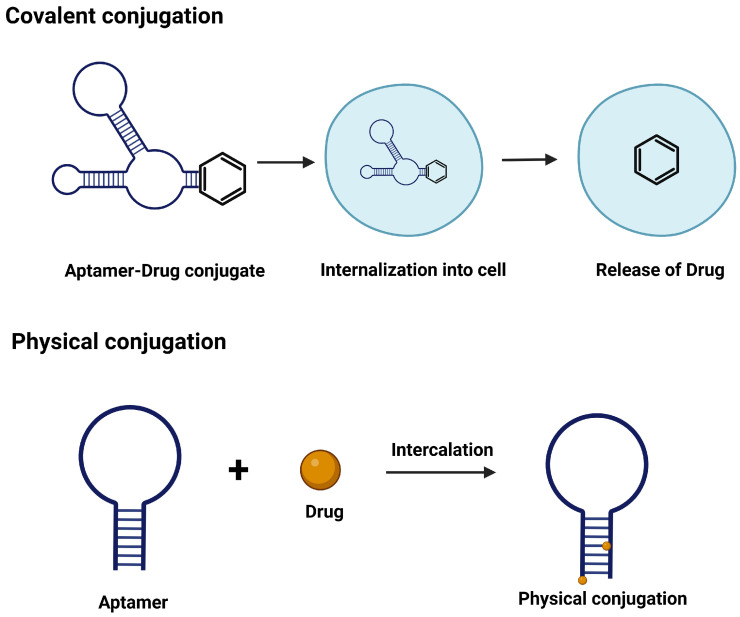
Mechanism of covalent and physical conjugation mechanisms common to aptamer–drug, aptamer–exosome, and aptamer–peptide systems, focusing on aptamer–drug conjugates. Created in BioRender. Gangadaran, P. (2026) https://BioRender.com/wjhgkwb, accessed on 6 April 2026..

The integration of peptide decoration and aptamer decoration onto exosome surfaces has rapidly emerged as a pioneering direction in the design of next-generation targeted drug delivery systems (Figure 3). Figure 3 outlines key conjugation strategies for engineering aptamer–peptide–exosome systems, distinguishing between covalent and noncovalent approaches. Covalent methods, such as EDC/NHS coupling and click chemistry, provide stable ligand attachment and prolonged circulation but may affect membrane integrity or ligand flexibility. In contrast, noncovalent strategies including hydrophobic insertion and electrostatic interactions, better preserve exosomal structure but offer reduced stability in physiological conditions. Thus, the choice of conjugation strategy critically influences targeting efficiency, biodistribution, and overall therapeutic performance [73,74]. To enable targeted delivery, peptides and aptamers can be attached to exosomal surfaces through covalent or noncovalent modifications. Covalent methods, including click chemistry reactions such as copper-catalyzed or strain-promoted azide-alkyne cycloadditions, allow stable attachment of therapeutic ligands, antibodies, nucleic acids, or peptides to the exosomal membrane, ensuring long-lasting functionality. Alternatively, noncovalent approaches rely on hydrophobic integration, electrostatic forces, or ligand–receptor binding to affix targeting molecules such as peptides and aptamers without compromising the delicate structural integrity or bioactivity of exosomes [7,75]. For example, exosomes decorated with RNA aptamers that bind to CD133, and angiopep-2 peptides have demonstrated effective crossing of the BBB and targeted delivery to glioblastoma tumors. Although minor accumulation in non-target organs still occurs, this highlights the emerging feasibility of this method to treat highly inaccessible and aggressive cancers [76].

The synergistic mechanism of peptide–aptamer conjugates can be exemplified through dual-decorated exosomes bearing the T7 peptide and the AS1411 aptamer. The T7 peptide sequence HAIYPRH binds transferrin receptor 1 (TfR1), which is abundantly expressed on brain capillary endothelial cells, thereby facilitating receptor-mediated transcytosis across the BBB. Meanwhile, the AS1411 aptamer, a G-quadruplex-forming oligonucleotide, binds to nucleolin, a protein that is overexpressed on glioblastoma cells but is minimally present in healthy tissues. The T7 peptide is genetically fused to exosomal membrane proteins, such as Lamp2b, for stable display. The AS1411 aptamer is covalently attached to surface amino groups using EDC (1-ethyl-3-(3-dimethylaminopropyl)carbodiimide)/NHS (N-hydroxysuccinimide) coupling chemistry, creating stable amide bonds between carboxyl and amine groups on the exosome surface. Upon systemic injection, this dual-ligand system first enables exosome crossing of the BBB. This then ensures tumor-specific uptake via caveolae-dependent endocytosis, whereupon therapeutic payloads, such as siRNAs, are released intracellularly to silence oncogenic targets. 

Alternatively, exosome platforms can integrate targeting peptides with aptamers connected via flexible molecular linkers to form peptide–DNA hybrid molecules. Targeting peptides recognize cancer-related receptors, such as EGFR or integrin αvβ3, while aptamers serve dual purposes: anchoring to exosomal surface proteins like CD63 or CD81 and hosting therapeutic cargo noncovalently via stem-loop structures that support π–π stacking and hydrogen bonding. This design preserves vesicle integrity as the hybrids noncovalently incorporate into purified exosomes. Upon administration, receptor-mediated uptake occurs, followed by intracellular cues such as acidic pH or enzymatic activity, which trigger controlled release of the therapeutic agent into the cytoplasm. This combined system also stabilizes fragile enzymes, such as catalase, and bolsters efficacy in hypoxic TMEs.

The vital link between peptides and aptamers on exosomes is underpinned by the peptide CP05, which binds specifically to the exosomal protein CD63. CP05 acts as a robust anchor, immobilizing exosomes without disturbing their morphology or size. Utilizing CP05 for exosome immobilization enables screening of specific DNA aptamers from extensive libraries via SELEX [77]. For effective therapeutic targeting, several critical criteria must be met [78].

**Figure 4:**
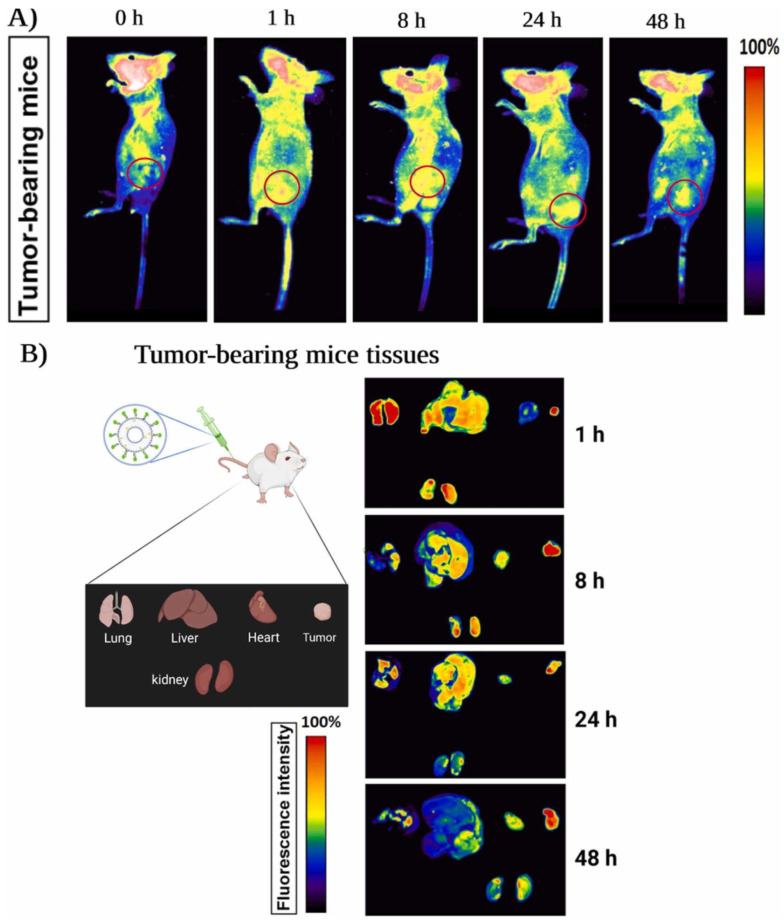
Biodistribution of FAM-labeled AS1411–exosomes in tumor-bearing mice. (**A**) Fluorescent images at 0 h (control) and after intravenous injection of AS1411-labeled exosomes; tumor regions are indicated in red. (**B**) Ex vivo imaging showing FAM-labeled AS1411 aptamer–exosome accumulation in the tumor, lung, liver, heart, and kidney at 1, 8, 24, and 48 h post-injection. (Adapted with permission from Hosseini et al., [72]; Copyright © 2022, The Author(s), CC-BY 4.0 International License).

This integrated aptamer–peptide–exosome platform offers several advantages over conventional antibody-based or small-molecule therapies. Peptides and aptamers are smaller, less immunogenic, and easier to synthesize and modify chemically for multiplex targeting. Aptamers can adopt dynamic conformations to precisely recognize evolving tumor epitopes. Peptides confer efficient cellular entry and tissue-specific targeting. Exosomes serve as native, stealthy carriers that can transport a variety of payloads, including RNA, chemotherapeutics, and proteins. This synergy culminates in highly effective, tunable nanocarriers that advance the goals of personalized cancer therapy while minimizing collateral damage [72,79]. Figure 4 demonstrates the in vivo biodistribution of AS1411-decorated exosomes, showing preferential tumor accumulation alongside measurable distribution in organs such as the liver and kidneys. This pattern highlights both the targeting capability of aptamer-decorated systems and the persistent challenge of off-target accumulation, underscoring the need for improved specificity and pharmacokinetic control. Clinically, aptamer- and peptide-decorated exosomes enable early, noninvasive cancer diagnosis through liquid biopsy, offering higher sensitivity by detecting exosomal miRNAs and other tumor markers. Therapeutically, the targeted, multimodal nature of these engineered exosomes promises safer cancer treatments with reduced side effects, overcoming biological challenges such as immune evasion and physiological barriers.

These in vivo observations (Figure 4) further reinforce the therapeutic potential of engineered exosomes; however, several translational challenges remain. While peptide- and aptamer-decorated exosomes represent a promising paradigm in targeted cancer therapy, several unresolved challenges limit their clinical translation. A critical issue lies in the discordance between preclinical efficacy and clinical scalability. The in vivo systems are significantly more complex, with factors such as protein corona formation, immune clearance, and tumor heterogeneity reducing targeting precision. Another major limitation is tumor heterogeneity, which undermines single-ligand targeting strategies [80,81]. Although peptides and aptamers exhibit high affinity toward specific receptors, heterogeneous expression of these targets across tumor subpopulations can lead to incomplete therapeutic coverage and potential relapse. This has prompted the exploration of multi-ligand or dual-targeting systems, though these approaches introduce additional complexity in design and validation. Furthermore, off-target accumulation and biodistribution variability remain contentious issues. Despite claims of high specificity, studies have reported accumulation of engineered exosomes in organs such as the liver and spleen, raising concerns about unintended biological effects and long-term safety [7,71,73].

## 6. Clinical Trials

Several peptide-based therapeutics, aptamer conjugates, and exosome-mediated delivery systems have advanced to preclinical and clinical evaluation for various cancers. These studies highlight the safety, specificity, and therapeutic potential of peptide and aptamer systems, while also revealing challenges related to bioavailability, immune response, and delivery optimization. Peptide drugs such as bortezomib, goserelin, and luteinizing hormone-releasing hormone analogs have already demonstrated clinical success, whereas newer agents, including peptide–drug conjugates and aptamer-decorated nanoparticles, are currently under investigation. Exosome-based therapeutics, though still in early stages, have shown encouraging results in terms of tolerability and tumor-targeted delivery.

Table 2 summarizes selected ongoing and completed clinical trials involving peptide-based, aptamer-based, and exosome-mediated therapeutics in cancer treatment.

## 7. Challenges and Future Perspectives

Despite significant progress in developing peptide-based therapeutics, several challenges continue to hinder their successful clinical translation. Significant limitations lie in their pharmacokinetic and stability profiles. Peptides are highly susceptible to enzymatic degradation, possess short plasma half-lives, and are rapidly cleared by renal filtration. Their poor membrane permeability further limits intracellular delivery and therapeutic efficiency [42,82]. Although various chemical and structural modifications, such as cyclization, PEGylation, and substitution with D-amino acids, have been employed to improve stability and bioavailability. These alterations may sometimes compromise receptor specificity or biological activity. Achieving an optimal balance between enhanced stability and preserved biofunctionality remains a key challenge in peptide therapeutics.

Large-scale production and standardization also present considerable obstacles. The synthesis of high-purity, biologically active peptides with proper folding and reproducibility is technically demanding and economically expensive. Similarly, the isolation and large-scale production of exosomes for therapeutic purposes are hindered by low yield and heterogeneity [83]. Conventional purification techniques, including ultracentrifugation and size-exclusion chromatography, often result in batch-to-batch variability and inconsistent cargo loading efficiency thus limiting reproducibility across studies. The lack of standardized protocols for exosome preparation, peptide conjugation, and characterization impedes reproducibility and hinders regulatory approval [84]. This variability complicates cross-study comparisons and raises concerns regarding experimental reproducibility and clinical reliability. Establishing universally accepted quality control measures and scalable production methods is essential for translating these technologies into clinically viable products.

Regulatory and ethical considerations further complicate the clinical adoption of peptide- and exosome-based therapies. The hybrid and biologically derived nature of these systems makes them difficult to classify within existing drug regulatory frameworks. Issues concerning long-term safety, immunogenicity, and potential off-target effects must be addressed through rigorous preclinical and clinical evaluations [85]. Additionally, the use of donor-derived exosomes raises ethical concerns regarding source traceability, consent, and biosafety. Clear regulatory guidelines and harmonized international standards are urgently needed to streamline approval processes and ensure patient safety [86]. From a regulatory perspective, peptide–aptamer–exosome systems exist at the intersection of biologics, nanomedicine, and drug delivery platforms, making classification ambiguous. Regulatory agencies require rigorous compliance with Good Manufacturing Practice (GMP), alongside comprehensive toxicological profiling and long-term studies on biodistribution and immunogenicity. Additionally, the use of donor-derived exosomes introduces ethical and safety concerns related to source variability, contamination risks, and traceability [12,86].

Emerging technologies are reshaping the landscape of peptide therapeutics. Artificial or biomimetic exosomes designed to mimic natural vesicles with improved scalability and structural control represent a promising alternative for targeted drug delivery [87]. Peptide–aptamer hybrid systems, which integrate the binding precision of aptamers with the functional diversity of peptides, are gaining traction as versatile therapeutic platforms. Moreover, the concept of personalized peptide therapeutics tailored to individual tumor profiles offers a new dimension in precision oncology [88]. Advances in computational modeling, artificial intelligence-driven peptide design, and exosome engineering are expected to address many current limitations. Together, these innovations pave the way for the next generation of safe, efficient, and patient-specific peptide-based cancer therapies.

## 8. Conclusions

Peptides, aptamers, and exosome-based delivery systems represent emerging tools in precision cancer therapy. Their high specificity, low toxicity, and tunable structures enable targeted modulation of tumor pathways and efficient drug delivery. Peptides and aptamers offer complementary advantages; while peptides act as potent therapeutic agents, aptamers provide remarkable target selectivity and stability. Exosomes further enhance therapeutic precision by serving as natural biocompatible carriers for these molecules. The combination of peptide-decorated and aptamer-decorated exosomes offers a promising strategy to overcome limitations in stability and targeted delivery. Although challenges in pharmacokinetics, large-scale production, and regulatory approval persist, advances in nanobiotechnology and bioengineering continue to bridge these gaps. Together, these systems pave the way for peptide-driven precision oncology, offering a new generation of safer and more effective cancer therapeutics. While these systems demonstrate remarkable potential, their successful clinical translation will depend on addressing fundamental challenges related to biological variability, scalable manufacturing, and regulatory standardization, underscoring the need for interdisciplinary efforts bridging bioengineering, clinical research, and regulatory science.

## Figures and Tables

**Table 1:** Summarizes the mechanism of action of various types of peptides in cancer.

Peptide	Type	Mechanism of Action
TAT	Cell-penetrating peptide	TAT-CPT and TAT-2CPT, formed by linking CPT to the TAT peptide that enhances TAT’s insertion into cancer cell membranes, creating pores, while released CPT inhibits topoisomerase I, triggering apoptosis [31].
BIM BH3	Inhibitory peptide	BH3 α-helical death domain, allowing it to bind and inhibit anti-apoptotic BCL-2 family proteins releases pro-apoptotic effectors, such as BAX and BAK, inducing mitochondrial outer membrane permeabilization and the release of cytochrome c [32].
NGR	Tumor-homing peptide	The NGR peptide targets tumor vasculature by binding to APN/CD13 receptors, which are overexpressed on tumor vessels and cancer cells, delivering attached drugs or probes directly [33].
Pep-1	Cell-penetrating peptide	Pep-1 facilitates liposome uptake by glioma cells. When carrying cilengitide, these Pep-1-decorated liposomes enhance targeted inhibition of integrins, thereby improving drug delivery efficiency [34].
F3	Tumor-homing peptide	The F3 peptide binds to nucleolin, which is overexpressed on tumor cells and vasculature, enabling targeted delivery and deep tumor penetration. When fused to TRAIL, it enhances the activation of death receptors, triggering apoptosis [35].
ALRN-6924	Inhibitory peptide	ALRN-6924 is a stapled peptide that mimics p53’s N-terminal domain, binding strongly to MDM2 and MDMX to block p53 degradation. This restores p53’s tumor-suppressive functions in TP53-wildtype cancers, triggering apoptosis [36].
Transportan-10	Cell-penetrating peptide	TP10 penetrates cancer cell membranes by translocating into the cell or disrupting the membrane. This membrane destabilization, leading to cytotoxic effects and facilitating internalization of therapeutic molecules [37].
Stapled BCL9 peptide	Inhibitory peptide	The stapled BCL9 peptide mimics BCL9’s helical domain, competitively binding β-catenin and blocking their interaction followed by reducing cancer cell proliferation and survival [38].
R8 (octa-arginine)	Cell-penetrating peptide	The R8-modified hydrogel selectively targets cancer cell membranes, enhancing drug uptake and penetration by promoting multiple internalization pathways, thereby increasing the effectiveness of chemotherapy while minimizing harm to normal cells [39].
CREKA	Tumor-homing peptide	The CREKA peptide targets fibrin-fibronectin complexes in the TME, enhancing nanoparticle binding and uptake by cancer cells. This increases localized drug delivery [40].
T7 peptide	Tumor-homing peptide	The T7 peptide induces apoptosis and cell cycle arrest in cancer cells by modulating apoptotic proteins and inhibiting the Akt/mTOR pathway [41].

CPT: camptothecin; TAT: Trans-activator of transcription; BIM BH3: Bcl-2-interacting mediator BH3; NGR: Asn-Gly-Arg; Pep-1: Peptide-1; F3: F3 Tumor-homing peptide; ALRN-6924: Stapled p53-mimetic peptide; TP10: Transportan-10; BCL9 stapled peptide: Stapled BCL9 peptide; R8: Octa-arginine; CREKA: Cys-Arg-Glu-Lys-Ala; T7: Tumor-homing peptide.

**Table 2:** Representative clinical trials of peptide-based and aptamer-mediated therapeutics in cancer.

Therapeutic Agent	Type	Target/Mechanism	Cancer Type	Clinical Phase/Status	Reference
Bortezomib	Peptide-based proteasome inhibitor	Induces apoptosis via 26S proteasome inhibition	Multiple myeloma, lymphoma	Approved (Phase IV)	FDA-approved
Goserelin	Peptide analog (LHRH agonist)	Suppresses LH and FSH secretion to reduce sex hormone levels	Primary peritoneal carcinoma, ovarian cancer	Approved	NCT00002960
CendR peptide (iRGD)	Tumor-penetrating peptide	Targets αv-integrins and neuropilin-1 to enhance tumor permeability and drug delivery	Metastatic pancreatic ductal adenocarcinoma	Phase I	NCT03517176
AS1411	DNA aptamer	Nucleolin-targeting; aptamer binds cell-surface nucleolin, internalized in tumor cells	Renal cell carcinoma, acute myeloid leukemia	Phase I completed	NCT00881244
NOX-A12	Spiegelmer (L-RNA aptamer)	Inhibits CXCL12/SDF-1–CXCR4 interaction to disrupt microenvironment signaling and cell trafficking	Relapsed multiple myeloma	Phase II ongoing	NCT01521533
exoASO-STAT6	Engineered exosome carrying antisense oligonucleotide	Silences STAT6 in tumor-associated macrophages to shift M2 → M1 phenotype and boost antitumor immunity	Advanced hepatocellular carcinoma, gastric cancer, colorectal cancer	Phase I	NCT05375604
GP2 and AE37 peptides (with GM-CSF adjuvant)	Peptide vaccine	HER2/neu-derived peptides designed to elicit cytotoxic T-cell responses against HER2-expressing tumor cells	Breast cancer (node-positive or high-risk node-negative)	Phase II completed	NCT00524277
E75 (Nelipepimut-S)	Peptide vaccine	Elicits cytotoxic T-cell response against HER2/neu-expressing tumor cells	Breast cancer	Phase III completed	NCT01479244

Bortezomib: Peptide-based proteasome inhibitor; Goserelin: LHRH agonist peptide analog; LHRH: Luteinizing hormone-releasing hormone; LH: Luteinizing hormone; FSH: Follicle-stimulating hormone; CendR peptide (iRGD): Tumor-penetrating peptide; AS1411: DNA aptamer; NOX-A12: Spiegelmer (L-RNA aptamer); exoASO-STAT6: Engineered exosome carrying antisense oligonucleotide; GP2: HER2/neu-derived peptide vaccine; AE37: HER2/neu-derived peptide vaccine; HER2: Human epidermal growth factor receptor 2; E75 (Nelipepimut-S): HER2/neu-targeted peptide vaccine.

## Data Availability

No new data were created or analyzed in this study.
